# Primary Care Providers’ Views of Patient Portals: Interview Study of Perceived Benefits and Consequences

**DOI:** 10.2196/jmir.4953

**Published:** 2016-01-15

**Authors:** David P Miller Jr, Celine Latulipe, Kathryn A Melius, Sara A Quandt, Thomas A Arcury

**Affiliations:** ^1^ Wake Forest School of Medicine Department of Internal Medicine Winston-Salem, NC United States; ^2^ College of Computing and Informatics Department of Software and Information Systems University of North Carolina at Charlotte Charlotte, NC United States; ^3^ Wake Forest School of Medicine Department of Family and Community Medicine Winston-Salem, NC United States; ^4^ Wake Forest School of Medicine Department of Epidemiology and Prevention Winston-Salem, NC United States

**Keywords:** vulnerable populations, personal health records, primary health care, attitude

## Abstract

**Background:**

The United States government is encouraging physicians to adopt patient portals—secure websites that allow patients to access their health information. For patient portals to recognize their full potential and improve patient care, health care providers’ acceptance and encouragement of their use will be essential. However, little is known about provider concerns or views of patient portals.

**Objective:**

We conducted this qualitative study to determine how administrators, clinic staff, and health care providers at practices serving a lower income adult population viewed patient portals in terms of their potential benefit, areas of concern, and hopes for the future.

**Methods:**

We performed in-depth interviews between October 2013 and June 2014 with 20 clinic personnel recruited from health centers in four North Carolina counties. Trained study personnel conducted individual interviews following an interviewer guide to elicit perceptions of the benefits and disadvantages of patient portals. Interviews were recorded and transcribed. Research team members reviewed transcribed interviews for major themes to construct a coding dictionary. Two researchers then coded each transcript with any coding discrepancies resolved through discussion.

**Results:**

The interviews revealed that clinic personnel viewed patient portals as a mandated product that had potential to improve communication and enhance information sharing. However, they expressed many concerns including portals’ potential to generate more work, confuse patients, alienate non-users, and increase health disparities. Clinic personnel expected few older and disadvantaged patients to use a portal.

**Conclusions:**

Given that clinic personnel have significant concerns about portals’ unintended consequences, their uptake and impact on care may be limited. Future studies should examine ways portals can be implemented in practices to address providers’ concerns and meet the needs of vulnerable populations.

## Introduction

Electronic health records can reduce medical error and enhance efficiency, particularly by facilitating the sharing of medical information [1]. Many electronic health records include patient portals—secure websites where patients can access their health information, request medication refills, and even communicate electronically with their health care provider. Recognizing the potential benefits of electronic health records and patient portals, the United States government is encouraging their adoption. In 2009, Congress passed the Health Information Technology for Economic and Clinical Health (HITECH) Act, which authorized incentive payments to physicians who demonstrated “meaningful use” of these new systems [2]. Beginning in 2015, providers who fail to adopt these new technologies will be penalized a small percentage of their Medicare reimbursements [3].

The Centers for Medicare and Medicaid Services is responsible for developing the “meaningful use” criteria, which they are releasing in stages. The initial meaningful use criteria included items entirely under health care providers’ control, such as giving visit summaries to patients or electronically sending medication refills to pharmacies. However, the latest round of criteria, released in July 2014, included items that require patient engagement, such as specifying that at least 5% of patients access their health information through a patient portal [4].

In large health care systems that have implemented patient portals, the initial response from patients has been tepid. A 2-year study found that only 10% of veterans had authenticated their patient portal account within the Veterans Health Administration system [5]. Even in large commercial health systems, typically less than 30-40% of patients activate their online access [6-8]. In addition, older patients and those from vulnerable populations are even less likely to use a patient portal [7,9,10]. In clinics serving primarily disadvantaged populations, portal use has been less than 10% [11]. For patient portals to recognize their full potential and improve patient care, health care providers’ acceptance and encouragement of their use will be essential [12-14]. However, little is known about providers’ concerns or views of patient portals.

We conducted this study to learn from the early experiences of clinics that have implemented or are in the process of implementing a patient portal. All clinics served a lower income population, allowing us to specifically examine issues related to vulnerable groups. We particularly wanted to determine how administrators, clinic staff, and health care providers viewed patient portals in terms of their potential benefit, areas of concern, and hopes for the future. Knowing this information could help health care systems optimize their use of patient portals, leading to improvements in patient care and fulfillment of meaningful use criteria.

## Methods

Data collection was completed between October 2013 and June 2014. We conducted this study as part of a larger multi-component investigation of factors that facilitate or hinder the use of patient portals among low-income older adults [15]. The project is a collaboration of a large academic medical center, a state university, and a network of 16 health centers located across rural North Carolina. The project protocol was approved by the Wake Forest Baptist Health Institutional Review Board, and all participants provided signed informed consent.

### Participants

We recruited 20 participants from health centers in four North Carolina counties representing variation on the urban-rural continuum [16]. The health centers also represented diversity and included an urban academic health center and three rural federally qualified health centers. To identify the full spectrum of barriers and facilitators of patient portal implementation, we purposefully recruited participants who had varying experience with portals ranging from current experience to prior experience to no experience. Investigators selected potential participants from the health centers to reflect a desired diversity in job category and geographic distribution. While it is suggested that 12-15 interviews are generally adequate for qualitative research [17,18], the number is ultimately determined by the researchers. Based on this study teams’ experience, 20 interviews would provide ample data to accurately describe health care providers’ experiences with patient portals across various study sites.

### Data Collection

Three trained interviewers completed one-on-one in-depth interviews with each participant. Interviewers met participants at a location of the participants’ choice, usually their office or clinic. Following each interview, participants received a small incentive (US $20) to thank them for their time. All interviews were audio recorded and transcribed for later analysis. Project staff contacted potential participants until the desired number of participants was achieved. Saturation was reached as it was determined that variability within the dataset had been achieved and no novel information was being gathered.

### Interview Content

The interview was designed to elicit use of technology and electronic patient information management systems by health care providers and to understand their perception of patient use of patient portals (see Multimedia Appendix 1). First, participants were asked about the use of patient portals in their practices, including if their practice had implemented a patient portal, the observed or anticipated impact of a portal on providers and patients, and the anticipated advantages and disadvantages of using a patient portal. Second, participants were asked about any privacy or security concerns regarding electronic personal health information and concerns their patients had about using the patient portal. Third, participants were asked about the environmental and community factors that impact use of patient portals, including facilitators and barriers for practitioners and patients.

### Analysis

Data analysis was based on a systemic, computer-assisted approach [19]. Mechanics of data management were accomplished through the use of ATLAS.ti (Scientific Software Development GmbH). All interviews were transcribed verbatim, and each transcript was edited for accuracy. Data analysis began with the collection and ongoing reflection on interview content through listening to interview recordings and reading the interview transcripts. Research team members reviewed each interview and recorded themes, patterns, and issues that arose in those narratives [20].

The entire research team discussed this information and developed a coding dictionary to reflect themes present in the interviews as structured according to Davis’ Technology Acceptance Model (TAM) [21]. Based on Fishbein and Azjen’s Theory of Reasoned Action and Theory of Planned Behavior [22], TAM posits the belief-attitude-intention-behavior causal relationship for predicting acceptance of information technology. Two beliefs are fundamental determinants of technology use: perceived usefulness and perceived usability. Perceived usefulness is “the degree to which a person believes that using a particular system would enhance his or her job performance,” while perceived usability is “the degree to which a person believes that using a particular system would be free of effort” [21]. Further, perceived attributes are important: users are more likely to use technology if the applications involved are both easy to use and meet users’ values and needs [23,24].

During an initial training period, the research team members practiced coding interviews to reach agreement on assignment of codes. Each transcript was coded by 2 research team members, and any differences were resolved through discussion. This double coding throughout data processing was a check on completeness and drift from the original code definitions.

## Results

Study team members contacted 30 health care providers to reach our target sample of 20 participants. Among the 10 non-participants, one refused to participate for lack of interest, 3 did not respond, and 6 were added to a waitlist. The 20 participants represented a range of positions from the 4 health centers (Table 1).

Approximately one-third of participants worked at clinics that were planning a new portal implementation to replace a prior portal that was discontinued for lack of use. The prior portal required an email address to register, and few of these rural clinics’ patients had email accounts. The planned new portal does not require an email to register.

**Figure 1:**
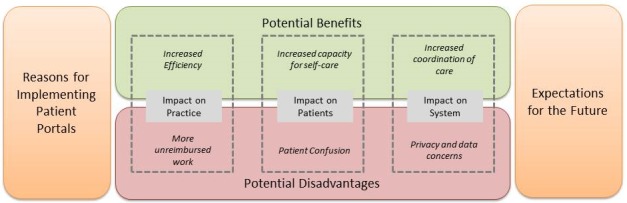
Categories of major findings from interviews conducted with clinic personnel.

Interviews with each participant ranged from approximately 30 minutes to 2 hours in length. Following the structure of our interviewer guide, we organized our findings along four broad categories as displayed in Figure 1. Within the categories of “potential benefits” and “potential disadvantages,” themes emerged detailing factors that primarily impacted the clinic, the patients, or the larger health care system.

**Table 1:** Characteristics of the study sample (N=20).

Characteristics		Value
**Clinic position, n (%)**		
	Nurse	6 (30)
	Physician/advanced practice provider	8 (40)
	Other non-medical clinicians	2 (10)
	Clinic manager/administration	4 (20)
**Clinic location, n (%)**		
	Rural	10 (50)
	Urban	10 (50)
Female, n (%)		15 (75)
Age, mean (range)		44 (24-70)
**Race, n (%)**		
	White, non-Hispanic	9 (45)
	African American	4 (20)
	American Indian	3 (15)
	Other	4 (20)
**Availability of patient portal at clinic site, n (%)**		
	Active patient portal in place	6 (30)
	Had portal previously; planning new portal implementation	7 (35)
	Never had portal; planning first time portal implementation	7 (35)

### Reasons for Implementing Patient Portals

The main motivator for implementing patient portals was external pressure, and in particular, new federally mandated requirements for “meaningful use” of electronic health records. We heard concern about being “rated” on portal use in the future. As one urban physician stated, “So it’s going to be an issue of ‘How many of your patients are using the patient portal?’ Two. ‘Oh? Two? Well, you failed’.” Similarly, a rural clinic administrator offered, “It’s just one more thing that you have to do, and it’s mandated. You’ve got to do it.”

### Potential Benefits of Patient Portals

While feeling pressured to adopt patient portals, staff and providers see several potential benefits to their use. These benefits are listed in Table 2 and described in further detail below.

**Table 2:** Potential benefits of patient portals identified by clinic personnel.

Potential benefit		Representative statements
**Office efficiency**		
	Decreasing phone calls	Clinic staff: “If there’s a lot of questions that can be answered through the portal that may cut down on even patients having to call in…” Nurse: “If somebody calls just for a prescription refill, they can do that online and save that phone call for somebody that really, really needs it.”
	Handling messages more quickly	Physician: “It’s also a lot easier just to type a message, because I could reply to an email in a few seconds, versus having to sit down, open a chart, pick up the phone, call, hope they answer, and if they don’t, having to call back later.”
	Eliminating need to inform patients of normal results	Physician: “There are a lot of steps in the process right now of notifying patients to let them know of their lab results. So if a patient were able to access that without us having to go through so many steps, that would be nice.”
**Patient/caregiver access to information**		
	Increasing patient ability to manage their health	Clinic staff: “they would get more information,...they would understand more, they would be more informed.”
	Increasing caregiver ability to assist with medical affairs	Nurse: “One patient was telling me that their family member was out in California, I think, a long way off, and she was her power of attorney but she couldn't get out there every time she needed to go to the doctor…but she was able to see what took place at the doctor’s office, what exactly was going on with her, and she said that helped her make decisions to benefit the family member instead of make the wrong decision.”
	Increasing patient satisfaction	Nurse: “So I had talked to a couple of patients that had actually gotten on it. They was going crazy. ‘I love it. I love being in this,’ because they feel like they have some charge on their own health.”
**Information sharing with other health professionals**		
	Decreases duplicate tests	Physician: “I mean tests are so duplicated when people go to different providers. It’s just ridiculous. And so if you had a patient who was going to their hematologist, and they were like, ‘Okay, I already got my CBC done at my primary,’ they could just go, ‘Here it is,’ and have a look at it.”
	Reduces medical error	Physician: “it’s good because it will allow less miscommunication between providers and less medical errors and less medication errors.”

#### Improving Office Efficiency

Many staff thought portals could improve office efficiency and save time, particularly by decreasing the volume of incoming phone calls for prescription refills and lab result requests. Nursing and clinic personnel can also triage and address electronic messages sent via a portal more quickly and efficiently than a telephone message. As one clinic administrator noted, “[nurses] get those messages on the computer where they’re sitting anyways. So they can quickly see them, cipher through what the patient said and actually try to get them an answer even before they call them back.”

Portals may have other unique benefits for clinics where providers are not present on a daily basis, such as resident clinics. For these settings, portals give providers immediate access to patients’ electronic requests, rather than requiring providers to physically return to the clinic to check a mailbox. Similar to nursing staff, physicians can also respond to electronic messages faster than phone messages. In addition, giving patients online access to their information reduced the need for staff to notify patients of routine results.

#### Improving Patient/Caregiver Access to Information

Similarly, portals can save patients time by giving them access to their health information: “Depending on their jobs or situation it may be easier for them to just quickly log on and check their information than having to make a phone call” (Clinic staff member). This feature was seen as particularly helpful for requesting prescription refills or making appointments, both of which can be done via a portal.

Additionally, staff expect portals to help patients better manage their care by providing them with easy access to their lab results, medication lists, and visit summaries. This feature could particularly benefit older adults who may have trouble understanding information during the medical encounter. One clinic administrator commented, “If [older adults] are having difficulty hearing or remembering what the doctor said, they can look it up.” Granting family members and caregivers access to patients’ medical information was seen as another benefit of portals.

#### Increasing Patient Satisfaction

If these benefits are realized, staff expect patient satisfaction and trust with the practice would improve. Clinic personnel observed that early adopters of portals greatly enjoyed the increased access to their health information. As one clinic administrator observed, “there’s something about that that gives you power and control. And everybody likes to know that they’re in control.”

#### Improving Information Sharing With Other Health Professionals

Improving patients’ access to medical records can also improve information sharing with other physicians, potentially improving care and decreasing duplicate tests. This improved information sharing could potentially result in fewer medical errors caused by patients’ not knowing their medication list, duplicate prescriptions, or incorrect therapies.

### Potential Disadvantages of Patient Portals

Despite these potential benefits, staff expressed many more concerns about the negative impacts of portals on their practices (Table 3). In general, their concerns can be categorized into threats to the practice, threats to patients, and threats to the health care system.

**Table 3:** Potential disadvantages of patient portals identified by clinic personnel.

Potential disadvantage		Representative statements
**Threats to practice**		
	Potential for high volume of messages	Nurse: “If the patients don’t wanna wait, they’ll email a hundred times or they’ll call a hundred times.”
	New time pressures	Clinic Staff: “We’re taking a phone call away but we just added an everyday procedure new thing that has to be done by the nurse that’s already overwhelmed.”
	Decrease in office visits	Nurse: “I saw that a patient thought, ‘Well, since I’m doing this now I don’t need to come to you as much,’ and that’s fine, but don’t miss your appointments. That part was not so good for us.”
	Liability concerns	Physician: “you have to be very careful about what you write, how you write, and what you’re telling the patient.”
**Threats to patients**		
	Causing patient confusion/anxiety	Physician: “If a patient had all of that data right in front of them without understanding which values may or may not be important that could just lead to unnecessary confusion; whereas if you just…go over results in person with a patient or just send a result card saying your labs are normal you don’t have to go into that level of confusion.”
	Alienating older patients	Nurse: “But as far as my older population – when I say “older” I mean 65 and up – I think that it’s gonna be a challenge, because they don’t understand...And a lot of them don’t really care about that stuff [computers]. I mean, when they’re 65 and older they come here, they want me to tell them, and that’s it.”
	Widening health disparities	Physician: “This is actually going to create a gap between people that are educated and have private insurance, they can have easier access to health and health questions, and people that aren’t – the barriers are just going to be bigger.”
**Threats to system**		
	Inaccurate data entry	Clinic Administrator: “And they understand that a human error could put some misinformation in there. And then they’ll say, ‘Well how’re you gonna get it out?’ And that’s a good question you know, that does not happen easy.”
	System failures	Clinic Staff: “I guess like anything technology is not perfect so if there were to be any glitches – anything can happen…but of course with the Internet and with anything there’s going to always be complications.”
	Privacy concerns	Physician: “People could potentially have medical information leaked through it. If they don’t use a strong password on their account, it’s certainly possible for someone to gain access to their information.”

#### Threats to Practice

While some staff saw the potential for portals to improve office efficiency, many more comments were made about how portals could hamper workflows and increase stress. Several nurses and physicians feared some patients would inappropriately send repeated messages, overwhelming clinic staff. One physician stated, “I’ve heard of other colleagues who have had patients who maybe sort of abuse it, and write a little too many emails back and forth, and are just – you know, it’s one question after the next after the next after the next.”

The potential demands portals could place upon scarce time was a theme in several interviews. One physician worried that patients may expect immediate responses to their electronic requests. Nursing staff were also concerned about the extra tasks patient portals would introduce into their days. Just the task of informing patients of the portal was viewed as a burden. One nurse in a clinic explained,

It’s hard to take the time with them patients and show ’em—“This is what you have to do, this is what it is”—because we have like 5 other patients waiting for that provider in that room. Our goal is to get everybody out to lunch and then everybody off on time so there’s no overtime.

Portals also may inadvertently decrease the perceived need for office visits. As one physician explained, “Like right now there’s a problem in medicine that people want all their care over the phone, and this just adds another layer to ‘I want all of my care for free’.”

We observed some disagreement about whether patient portals would increase or decrease liability risk. One physician cited the risk of a privacy breach and a large monetary fine if a computer containing patient communications were lost. Another physician viewed electronic exchanges in the portal as being held to the same standard as an office visit and cautioned, “You have to also be careful about the information that you send because any information that you send is like seeing a patient.” Conversely, another physician felt portals would protect physicians by saving “a perfect record of the entire conversation.” If a patient were to later complain, this physician believed these electronic “records could just be given and everyone would know exactly what transpired” rather than having to “go to court.”

#### Threats to Patients

Some staff and providers were concerned that patients would not fully understand the information present in the portal, triggering more phone calls and questions. Physicians remarked that lab results could be particularly troublesome because clinically insignificant abnormal results are common. Providers also felt their older patients would not want to use the portal and may feel alienated from the practice if they do not. As one administrator stated, “for younger more tech savvy patients, it’s awesome and they think it’s great.” However, older patients could “feel a little left behind” as practices implement portals. One nurse especially saw this concern for patients who lacked literacy or technical skills:

Every time they come they have the same question, the same problem, and they’re just not understanding it and it makes them feel not wanting to come here if somebody’s gonna be pushing something like that [the portal] on them. They feel like we’re pushing it.

Two physicians expressed concerns that patients with insurance, higher education, or better access to technology would benefit from the additional services of patient portals while older or vulnerable patients who do not use portals would become further disadvantaged. For patients who fail to use a portal, “the barriers are just going to be bigger” (Physician).

#### Threats to System

Several staff and providers expressed concerns about the stability of new technology and security of information. Administrators and staff acknowledged the inevitability of user error and the potential for incorrect information to be entered into charts. New technology was seen as prone to technical bugs and breakdowns. Last, there was a general concern for the security of information on the Internet. Any new portal was seen as “a potential information leak” (Physician) that could occur through a number of means: a stolen password, a shared password, or hackers (Physician and Staff at three separate sites).

### Expectations for the Future

#### Low Expectations for Immediate Use

We found general agreement among staff and providers that few patients would use a patient portal. For one physician, this low uptake of the portal was seen as a reality: “Let’s put it this way, I saw a patient with a resident earlier last week, and it actually said that they have an active [patient portal] account, and I was surprised. That’s how infrequently I see people that have it.” An administrator in a different county described her experience with a prior attempt to launch a patient portal: “even with our effort, there was nobody who actually used it after we had about 100 sign up.”

The low uptake of portals by patients discouraged providers from using the portals as well: “Because a lot of my patients haven’t signed up for it, I don’t use it to communicate systematically with them. I don’t think of sending them letters or communicating with them on [the portal]” (Physician). Similarly, a nurse with prior experience with a portal stated, “they wanted us to check it every day and that type of thing. As I checked it, like I said, it was the same thing the whole time. I just stopped checking it.”

#### Higher Expectations for Future Use

Although staff and providers viewed current use of patient portals as being very low, they had greater expectations for the future. Because patient portals are still a relatively new technology, some envisioned that usage would increase in the future as the population changes but predicted it would take several years to see uptake increase significantly (Clinic administrator). One clinic staff member compared the current use of portals to the early days of electronic banking: “Like ATMs and banking I think there would be a transition period where some people are still going to go inside and want that.”

### Rural/Urban Similarities and the Digital Divide

We found similar attitudes about the barriers to implementing patient portals in rural and urban clinics, as well as in clinics with a current active portal compared to those with no portal. Rather than a geographic digital divide, we observed a divide defined by age, education, and income. In particular, older adults were viewed as lacking the skills to operate a computer or smartphone, limiting their access to the Internet. This lack of computer literacy was attributed in part to a general anti-technology attitude among the elderly. As examples, one nurse stated “a computer scares them to death” and a physician remarked “[they] don’t typically like computers, even if they have one.”

All of the clinics in our sample served socioeconomically disadvantaged patient populations. In these clinics, providers believed a large number of their patients lacked the education to know how to use a computer or the income to afford home access. In some clinics, staff estimated that half the patients had no home Internet connection. The free Internet access provided in libraries was viewed as a poor substitute for home access: “If you are sitting in your house and you have a question about your medical record, if the practice is open, you’re going to call. Are you really going to get in the car and drive over to the library?” (Clinic administrator).

In contrast, clinic providers felt “[patient portals] are a good idea in the private practice setting, because you have people who have smartphones, you have people who are knowledgeable, you have people who know how to navigate them.”

## Discussion

### Principal Findings

By interviewing staff and providers from a variety of health centers, we were able to gather “front line” views of the early stages of implementing a patient portal. We purposively recruited clinics that serve a low-income population, allowing us to identify issues relevant to vulnerable populations. Clinic personnel in our study identified some benefits to portals, such as their potential to improve communication, give patients easy access to information, and enhance information sharing. However, we heard many more concerns about portals’ potential to generate additional work, confuse patients, and perhaps alienate non-users. In general, staff saw patient portals as a mandated product that will rarely be used by older adults. This, in turn, discouraged providers from embracing this new technology. Perhaps because providers expected few patients to actually use the portal, we heard very few concerns about the potential for portals to shift reimbursable office visits to un-reimbursable electronic exchanges.

While this study is one of the first to investigate practices’ early experiences with patient portals, a few other studies have explored physicians’ thoughts about allowing patients to view health records or communicate electronically with clinicians. Similar to our findings, the majority of physicians believed implementing a patient portal would increase their workload [25,26]. Likewise, practice managers and physicians who use electronic communication with patients agree that it creates more work and adds pressure to their day [27]. In the interviews we conducted, staff and physicians worried that some patients may abuse a portal’s easy access to providers. Because electronic patient-provider messaging is a recent development, practices should define clear expectations for appropriate use to guide patients and minimize misuse until new cultural norms emerge.

Many clinicians in our study feared the information in portals could confuse patients causing concern and more calls to the clinic. Some health systems have granted their patients access to view their health records online. In these systems, the majority of physicians shared these concerns, yet fewer than 20% of patients agreed [28]. Another small study of primary care residents and faculty at a single academic institution found that after a portal was implemented, only 13% felt their workload had increased [25]. Although low portal uptake may have contributed to the minimal change in workload observed, these and other studies still suggest that clinicians’ fears of patient confusion and increased messages may not come to fruition [29,30].

Providers in our study believed few older adults would use a portal, a belief supported by a study in a large managed care organization reporting that the oldest adults were the least likely to log on to their portal [7]. An age-related digital divide may partially explain this finding. In national surveys, adults in the oldest age groups are the least likely to use the Internet or email [31,32]. In addition, those with functional impairments are also less likely to use the Internet [32,33]. Not surprisingly, patients without home computers and patients who do not use the Internet are less likely to register for a patient portal [6,34,35].

Compounding this issue, patients often rate portals as difficult to use and not user friendly [36]. A recent evaluation of three currently available personal health records found the majority of low socioeconomic status adults had difficulty navigating and using the systems, frequently requiring assistance [37]. Other analyses have found that members of vulnerable populations including those with less education, lower income, and low health literacy are the least likely to use the Internet or enroll in patient portals [7,8,10,13,14,32,38]. Despite this relatively low use of patient portals, interest in portals and electronic communication is often higher among racial/ethnic minorities and those with chronic medical conditions [13,39]. Still, if efforts to reach out to vulnerable populations fails to occur, health care disparities could increase as portal adopters reap the benefits of easier access to information leaving non-adopters behind [40]. Indeed, several studies have found that portal use is associated with improved patient self-management of disease, better patient-provider communication, and use of preventive health services [36,38,41].

Federal meaningful use criteria currently require at least 5% of a practice’s patients to view, download, or transmit their health information electronically, and at least 5% of patients must send a secure electronic message [4]. The Centers for Medicare and Medicaid recently proposed softening these requirements to at least 1% of patients accessing their health information electronically and documenting that a secure electronic messaging system was enabled [42]. Given clinics’ concerns about their low-income patients’ willingness and ability to use a patient portal, we are in favor of these relaxed requirements.

One of the most common reasons cited by patients for not using a portal is lack of knowledge or motivation [43]. This finding suggests that educating patients about the portal could help lessen the digital divide and prevent health disparities from increasing. A challenge for health systems will be identifying who can provide this training. In our interviews, we consistently heard that both clinicians and medical staff lack the time to take on extra tasks. Future research should focus on strategies for increasing portal adoption in vulnerable populations. One study found that showing a promotional video during a clinic visit had a small effect on increasing portal registrations [44]. Other potential strategies could include using non-clinical staff as trainers, holding workshops for interested patients, and creating user-friendly online tutorials. In general, clinics that have used a planned, systematic implementation strategy have seen higher rates of portal uptake than clinics that rely on clinicians to inform and enroll patients [45]. However, the importance of having clinician and provider buy-in before implementation has also been highlighted [29].

### Limitations

Our study has limitations. Because we were primarily interested in learning how older and vulnerable adults use patient portals, we selected providers from clinics that serve a primarily disadvantaged population. The attitudes and barriers we identified may not be as prevalent in practices serving a higher socioeconomic patient base. Similarly, although we selected clinics from a mixture of urban and rural locations, all our study clinics are located in North Carolina. Different regions of the country may experience different barriers unique to their populations. Likewise, clinics that operate under different reimbursement structures, such as Health Maintenance Organizations, may view things differently, for example, a portal’s potential to encourage more out-of-visit communication.

### Conclusion

In conclusion, clinic staff from every health center in our sample recognized potential benefits to patient portals but were also concerned about the new work and confusion portals could bring. Uptake of portals was seen as very low, further discouraging providers from embracing them. Future studies should examine ways portals can be implemented efficiently in practices and strategies for increasing portal usage in vulnerable populations, including older adults. For portals to reach their full potential and meaningfully improve care, clinicians and patients will need to view them as a technology that adds value to care.

## Appendix

Multimedia Appendix 1 Health Care Providers Interview Guide.

## Abbreviations

HITECH: Health Information Technology for Economic and Clinical Health
TAM: Technology Acceptance Model
