# Supplementary material for: Primary Care Providers’ Views of Patient Portals: Interview Study of Perceived Benefits and Consequences
Source: J Med Internet Res. 2016 Jan 15;18(1):e8. doi: 10.2196/jmir.4953 (PMC4733220; doi:10.2196/jmir.4953)
Supplement: Multimedia Appendix 1 [file jmir_v18i1e8_app1.pdf]

## Appendix

## Maximizing the Impact of ePHIM in Low Income, Multiethnic Populations
## Health Care Providers Interview Guide

### Personal Characteristics

Please tell me a little about yourself.

You can start with your age.

Where are you from originally?
How long have you been in this community?

Tell me about your education and training.

Tell me about your current work

How would you best characterize your practice?

Approximately how many providers are associated with this practice?

What is your current position?

How long have you been working at your current place of employment?

Do you directly care for any patients in your work?

What other work have you done in the past?

**Technology Background and Experience**

Use of Patient Portal

Now I'm going to ask you some questions about the use of patient portals in this practice.

Does your practice currently offer a patient portal?

**[If NO]**

What are the plans for installing a new patient portal in the future?
How do you feel about the use of a patient portal in this practice?
What do you think are some of the benefits of a patient portal?
What do you think are some of the disadvantages of a patient portal?

**[If YES]**

In which year did your practice install the current patient portal system?

What is the name of your current patient portal system?

What features does [ ] portal include?

How does [ ] portal change the daily processes in your practice?

Tell me about the benefits of [ ] portal for your practice.
Tell me about the disadvantages of [ ] portal for your practice.
Tell me about the benefits of [ ] portal for older patients.
Tell me about the disadvantages of [ ] portal for older patients.

Now I'm going to ask you some questions about your experience with using [ ] portal.

How long have you been using [ ] portal?
How comfortable are you using [ ] portal?
Tell me about how you use [ ] portal to communicate with older patients.
How does the [ ] portal change your interactions with clinic staff and other providers?
How does the [ ] portal change your interactions with older patients?
How does the [ ] portal help you engage older patients?
How does the [ ] portal help you care for older patients with complex conditions?
Tell me about how older patients value having [ ] portal?
What concerns do older patients have about using [ ] portal?
What limits older patients using [ ] portal?
What would make it easier for older patients to use [ ] portal?
What concerns do you have about using [ ] portal?

Privacy and Security

What concerns do older patients have about [ ] portal protecting their private health information?

What concerns do older adults have about the trustworthiness of information that they find on [ ] portal?

What concerns do you have about [ ] portal protecting the patient's private health information?

Environment and Community Factors

Tell me about how accessible [ ] portal is in this practice?

What limits the use of [ ] portal by providers and clinic staff in this practice?

What limits the use of [ ] portal by older patients in this practice?

What would help expand the use of [ ] portal by providers and clinic staff in this practice?

What would help expand the use of [ ] portal by older patients in this practice?

What would encourage older adults to access patient portals?

If you could change anything about [ ] portal, what would it be and why?
